# Central Lesions With Selective Semicircular Canal Involvement Mimicking Bilateral Vestibulopathy

**DOI:** 10.3389/fneur.2018.00264

**Published:** 2018-04-24

**Authors:** Luke Chen, G. Michael Halmagyi

**Affiliations:** ^1^Faculty of Medicine, University of New South Wales, Sydney, NSW, Australia; ^2^Neurology Department, Royal Prince Alfred Hospital, Sydney, NSW, Australia

**Keywords:** bilateral vestibulopathy, central vestibular disorders, semicircular canal, head impulse test, eye movements

## Abstract

Bilateral vestibulopathy (BVP), which is due to peripheral lesions, may selectively involve certain semicircular canal (SCC). Recent eye movement recordings with search coil and video head impulse test (HIT) have provided insight in central lesions that can cause bilateral and selective SCC deficit mimicking BVP. Since neurological signs or ocular motor deficits maybe subtle or absent, it is critical to recognize central lesions correctly since there is prognostic and treatment implication. Acute floccular lesions cause bilateral horizontal SCC (HC) impairment while leaving vertical SCC function unaffected. Vestibular nuclear lesions affect bilateral HC and posterior SCC (PC) function, but anterior SCC (AC) function is spared. When both eyes are recorded, medial longitudinal fasciculus lesions cause horizontal dysconjugacy in HC function and catch-up saccades, as well as selective deficiency of PC over AC function. Combined peripheral and central lesions may be difficult to distinguish from BVP. Anterior inferior cerebellar artery stroke causes two types of deficits: 1. ipsilateral pan-SCC deficits and contralateral HC deficit and 2. bilateral HC deficit with vertical SCC sparing. Metabolic disorders such as Wernicke encephalopathy characteristically involve HC but not AC or PC function. Gaucher disease causes uniform loss of all SCC function but with minimal horizontal catch-up saccades. Genetic cerebellar ataxias and cerebellar-ataxia neuropathy vestibular areflexia syndrome typically do not spare AC function. While video HIT does not replace the gold-standard, search coil HIT, clinicians are now able to rapidly and accurately identify specific pattern of SCC deficits, which can aid differentiation of central lesions from BVP.

## Introduction

Bilateral vestibulopathy (BVP) is a chronic vestibular syndrome defined by bilaterally impaired vestibulo-ocular reflex, variably involving semicircular canal (SCC) and otolith function (1), as typically assessed by individual SCC head impulse test (HIT) (2, 3) and vestibular evoked myogenic potential (4), respectively. Peripheral lesions, such as gentamicin vestibulotoxicity, autoimmune inner ear diseases, bilateral Meniere’s disease, and bilateral vestibular schwannomas are well recognized in BVP (5, 6). Central lesions, however, are increasingly recognized to affect SCC and otolith function bilaterally, thus potentially mimicking BVP (7–11). As neurological signs or other ocular motor finding may not be readily appreciable or rapidly evolving, it is important that central lesions are considered as a cause of BVP especially if a specific pattern of SCC involvement is apparent. Clinical or bedside HIT remains a useful screening test as no equipment is required and should be performed first before selecting patients for quantitative HIT such as search coil HIT (scHIT) and video HIT (vHIT). While scHIT is the gold-standard for evaluating individual SCC function (3), it is time consuming and semi-invasive, and, with the advent of modern video-oculography, rapid and reliable assessment of each SCC function is now possible in the clinic with vHIT (12, 13). In this review, we discuss current understanding of pattern of SCC abnormality in central lesions that can mimic BVP, drawing data from both scHIT and vHIT studies.

## Peripheral Lesions

Bilateral vestibulopathy is commonly defined by bilateral symmetrical horizontal SCC (HC) deficit (14), but also VEMP impairment (15), without clinical or radiological involvement of central vestibular structures such as brainstem and cerebellum. In severe BVP, the loss of function of all six SCC is usually total if not near total, and it is usually assumed that horizontal and vertical SCC function is equally affected. However, vHIT has shown that anterior SCC (AC) function may be selectively spared compared to posterior canal (PC) function in BVP due to gentamicin vestibulotoxicity and bilateral Meniere’s disease, whereas such sparing does not occur with idiopathic cases, those associated with sudden hearing loss and infection (11, 16). The mechanism of AC function sparing is thought to be disease specific. Isolated bilateral PC loss of function is an uncommon manifestation of BVP (17), often without an identifiable cause, and additional SCC deficits are usually unilateral rather than bilateral. Such cases would invariably be missed if only HC function is tested. Expectedly compensatory or catch-up saccade cumulative amplitude increases with decrease in SCC function so that gaze position error is minimized (14).

## Vestibular Nuclear Lesions

Isolated vestibular nuclear stroke may be clinically indistinguishable from acute peripheral vestibulopathy (18). In two cases of isolated acute vestibular nuclear stroke mapped to the medial vestibular nucleus on MRI, scHIT revealed bilateral HC and PC deficit while “skipping” AC (19). As AC afferents project to both superior and medial vestibular nuclei (20), selective lesions of medial vestibular nucleus would theoretically leave ipsilesional AC function intact. It should be noted that there is significant overlap of afferents to different parts of vestibular nuclei; in particular, HC afferents project to superior vestibular nuclei too (21). Contralesional HC deficit is possibly mediated by inhibitory interneuronal adaptive process, similar to the explanation for isolated floccular lesions (22). Thus, it may be impossible to differentiate isolated vestibular nuclear stroke from BVP with scHIT or vHIT, and correct identification should rest on the finding of additional eye movement abnormalities, such as direction-changing, gaze-evoked nystagmus, or skew deviation. Fortunately, isolated vestibular nuclear stroke is rare.

## Cerebellar Lesions

**Figure 1:**
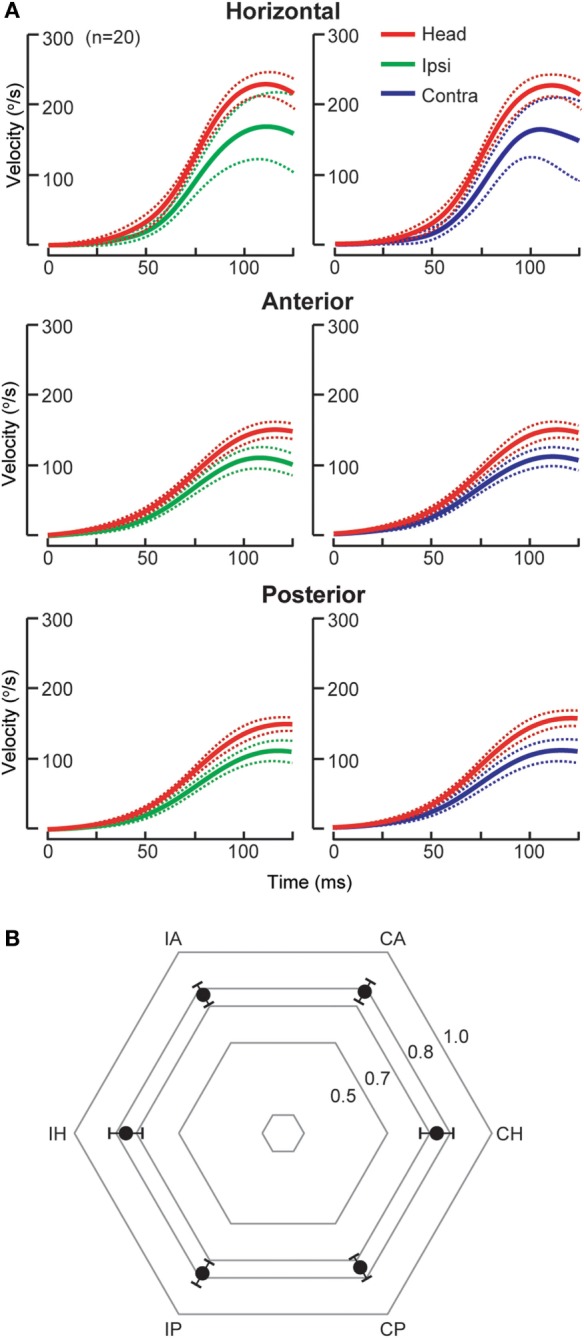
Individual semicircular canal (SCC) function in posterior inferior cerebellar artery (*n* = 17) and superior cerebellar artery stroke (SCA, *n* = 3). **(A)** Group data (mean, solid lines; 95% confidence interval, dashed lines) displayed as time series of inverted eye and head velocities during the first 125 ms of head impulses, recorded using search coil HIT (scHIT). SCC function is indicated by the gain, the ratio of eye to head velocity. There is mild bilateral HC impairment (gain: ipsilateral 0.75 ± 0.09, contralateral 0.74 ± 0.08), while both AC (gain: ipsilateral 0.76 ± 0.06, contralateral 0.78 ± 0.06) and PC function (gain: 0.77 ± 0.06, contralateral, 0.74 ± 0.06) is intact. Note for SCC function evoked by scHIT, the lower limit normal for gain is ~0.8 for HC and ~0.7 for vertical SCC. **(B)** Radar plot depicting group data (mean, solid circles; 95% confidence interval, bars) displayed as gain for each SCC. IH, ipsilesional horizontal canal; IA, ipsilesional anterior canal; CA, contralesional anterior canal; CH, contralesional horizontal canal; CP, contralesional posterior canal; IP, ipsilesional posterior canal.

Acute cerebellar lesions, whether unilateral or bilateral, can lead to selective moderate bilateral HC impairment sparing vertical SCC function (7, 23) as recorded by scHIT. This pattern is of potential diagnostic value as AC but not both AC and PC function is spared in peripheral lesions. Isolated unilateral floccular stroke, in the territory of anterior inferior cerebellar artery (AICA), causes bilateral HC deficit, slightly worse contralesionally, but discordant caloric and sinusoidal rotational responses (22). The unusual finding of unilateral cerebellar lesions causing bilaterally impaired HC function is thought to be due involve ipsilateral inhibitory floccular projection on the vestibular nuclei, the inhibitory vestibular interneurons and contralateral floccular adaption. The reason for sparing of vertical SCC function is unknown, perhaps related to preferential response of floccular Purkinje cells to vertical rotation (24, 25). Cerebellar stroke, mainly unilateral (70%) and involving the nodulus and uvula in the posterior inferior cerebellar artery (PICA) territory, causes mild reduction (25%) in HC function (7), but AC and PC function is not affected (Figure 1), as measured by scHIT. This is possibly due to preference for horizontal over vertical rotation of the eye-movement sensitive neurons, which consist of 20% of the nodulus target neurons, in the vestibular nuclei (26). The mechanism for lack of vertical SCC involvement is unclear, but is theoretically related to directional property of nodular/uvular Purkinje cells, which preferentially align with vertical SCC plane (27, 28). In contrast to BVP in cerebellar lesions, catch-up saccade cumulative amplitude does not necessarily correlate with SCC function. Compared to unilateral peripheral lesions, e.g., acute peripheral vestibulopathy, it is small during ipsilesional head impulses and may be larger during contralesional impulses (7). Dorsal vermal lesions can cause ipsilesional saccade hypometria (29) and the larger cumulative amplitude in cerebellar lesions might represent refixating eye movements in the face of saccade hypometria during contralesional impulses.

## Medial Longitudinal Fasciculus (MLF) Lesions

Lesions of the MLF disrupt binocular horizontal eye movement and causes the clinical syndrome of internuclear ophthalmoplegia: during horizontal volitional saccades, the abducting eye overshoots the target with or without nystagmus while the adducting slows and/or undershoots (30). Both horizontal and vertical SCC function is abnormal in MLF lesions, though there are some differences between bilateral and unilateral lesions (31, 32). In bilateral MLF lesions, horizontal SCC deficit is characterized by dysconjugacy in gain, as measured by the ratio of eye to head velocity, between the adducting and abducting eye, mirroring the dysconjugacy in horizontal volitional saccades: gain of the adducting and abducting eyes is both reduced, but is more seve-rely affected in the adducting eye (31). Several explanations have been put forward to account for abducting eye impairment, including additional abducens nerve or nuclear involvement (33) and impaired inhibition of antagonist medial rectus (34, 35). Impaired inhibition of medial rectus is unlikely to account for abducting eye impairment, since only excitatory but not inhibitory responses have been recorded when electrically stimulating the MLF (36). We have proposed another mechanism that could account for abducting eye impairment in bilateral MLF lesions: during impulses toward one MLF lesion, disfacilitation of the medial rectus motoneurons of the abducting eye, which normally receives excitatory input *via* the abducens interneurons traveling in the opposite MLF and is inhibited by type I vestibular neurons, is defective (31). Horizontal catch-up saccades are expectedly dysconjugate, in keeping with volitional saccade dysconjugacy. However, there is discrepancy between gain and catch-up dysconjugacy: despite little or no adducting eye catch-up saccades, there is partial preservation of adducting eye gain. The ascending tract of Deiter’s (37) is an extra-MLF pathway that mediates excitatory projection from vestibular nucleus to ipsilateral medial rectus motoneurons and could possibly account for the partial preservation of adducting eye gain. In unilateral MLF lesions, abducting eye gain deficit is absent, as disfacilitation of medial rectus motoneurons is intact *via* the unaffected contralateral MLF.

The pattern of vertical SCC deficits is similar for both bilateral and unilateral MLF lesions although the severity differs. PC contralateral to the MLF lesion is universally affected, consistent with all PC signals being transmitted *via* the MLF (38, 39). AC function is relatively less affected, due to the some AC signals being relayed through extra-MLF pathways such as the brachium conjunctivum and ventral tegmental tract (3). There is less AC function sparing in bilateral MLF lesions, possibly explained by three different mechanisms: relative strength of extra-MLF pathway for AC signal (40), on–off direction asymmetry in the vertical SCC plane (41, 42), and property of the vertical secondary vestibular neurons such as lower resting rate (43) and higher sensitivity to rotation (44).

The clinical implication is that there is potential for misdiagnosis of MLF lesions as BVP. All vHIT devices record monocular eye movements so that it will be not possible to determine horizontal gain dysconjugacy. Some vHIT devices measure only the right eye, whereas others can be adjusted to record either eye. For right eye systems, if there is a right MLF lesion, HC function will be deficient on rightward impulses, and there will be left PC deficit with some AC sparing. This should not be confused with a patchy BVP, as left HC function would be intact. If there is a left MLF lesion then both HC function would be intact, and the isolated AC–PC dissociation would be diagnostic. In bilateral MLF lesions without binocular eye movement recording, it would be easy to make the mistake of diagnosing BVP, as there would be HC deficit (due to adducting eye deficit from one MLF lesion and abducting eye deficit from impaired disfacilitation from the other MLF lesion), and variable bilateral AC-PC dissociation. Until binocular vHIT system becomes established, it is important to examine the horizontal saccades carefully.

## Combined Central and Peripheral Vestibular Lesions

It may be difficult to diagnose a combined central and peripheral vestibular lesion if only peripheral signs, e.g., SCC deficits consistent with BVP, or central signs, e.g., gaze-evoked nystagmus and impaired smooth pursuit are considered (45). However, certain patterns of SCC deficits and catch-up saccade characteristic should still prove helpful. Recent studies have highlighted a number of disorders that can present with combined central and peripheral lesions: AICA strokes and cerebellopontine angle (CPA) tumor (7, 23), metabolic disorders such as Gaucher disease(GD) (8, 46) and Wernicke encephalopathy (10, 47), and cerebellar degeneration (9, 48, 49). By far, over 70% are accounted for by AICA stroke and CPA tumor (45).

### AICA Stroke

**Figure 2:**
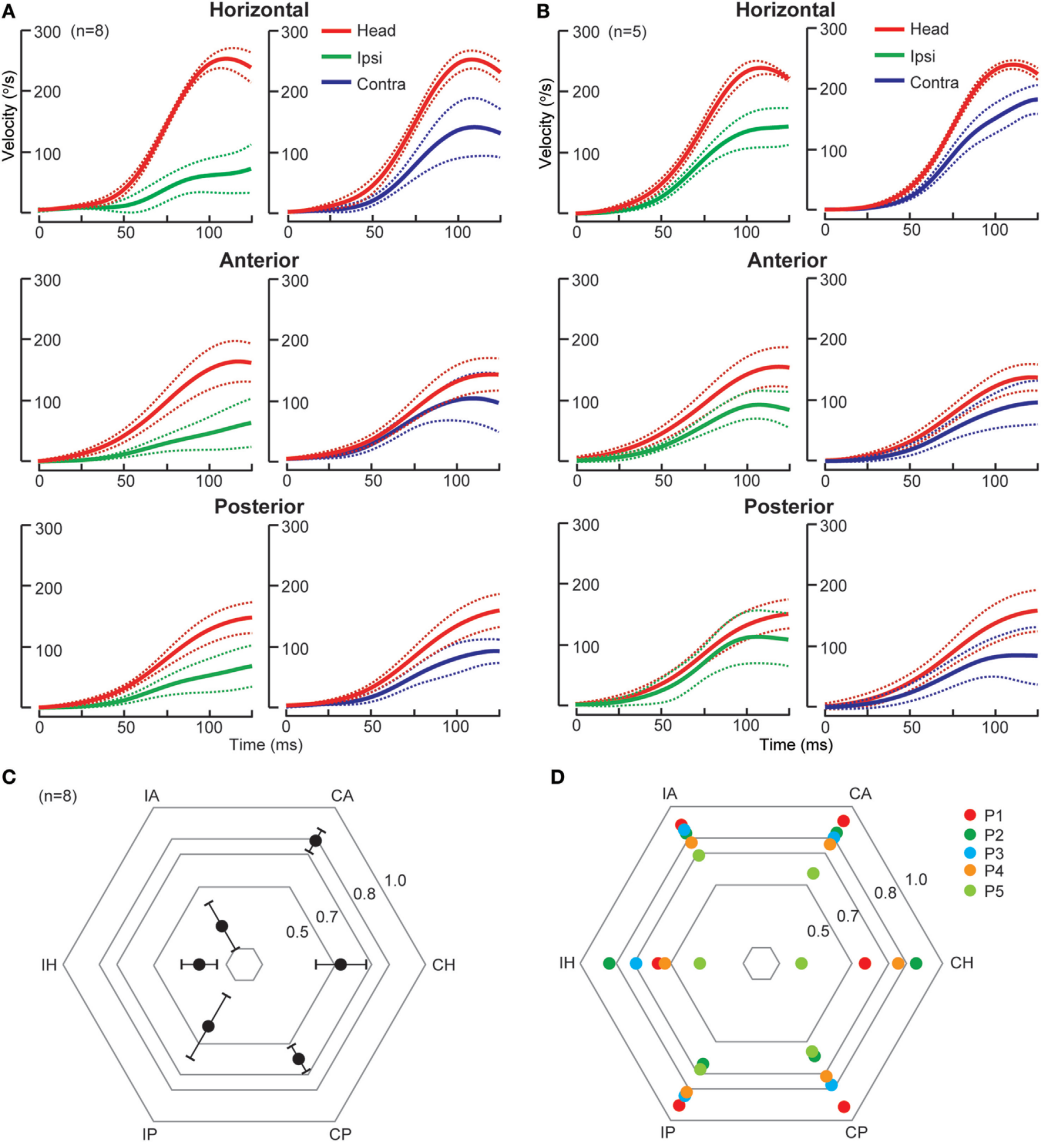
Individual semicircular canal function in anterior inferior cerebellar artery (AICA) stroke. **(A)** In the first type of AICA stroke (*n* = 8), group data displayed as time series of inverted eye and head velocities during the first 125 ms of head impulses, recorded using search coil HIT. Ipsilesional HC (gain 0.25 ± 0.10), AC (gain 0.24 ± 0.15), and PC (gain 0.39 ± 0.20) function was deficient, while only contralesional HC (gain 0.53 ± 0.14) but not AC (gain 0.79 ± 0.07) function was impaired. Contralesional PC function (gain 0.60 ± 0.08) function was slightly reduced, probably consistent with the severe ipsilesional AC deficit and reflects the on–off direction asymmetry. **(B)** In the second type of AICA stroke (*n* = 5), there is mild bilateral symmetrical HC deficit while AC and PC function is preserved. Radar plots depicting mean gain ± 95% confidence interval for the first type of AICA stroke (*n* = 8) and individual values for the second type of AICA stroke (*n* = 5) are presented in **(C,D)**, respectively. IH, ipsilesional horizontal canal; IA, ipsilesional anterior canal; CA, contralesional anterior canal; CH, contralesional horizontal canal; CP, contralesional posterior canal; IP, ipsilesional posterior canal; P, patient.

The AICA supplies the vestibulocochlear nerve, root entry zone, dorsolateral pons including the vestibular nuclei and flocculus (50). Thus, in AICA stroke, a spectrum of audiovestibular loss is expected (51). There are two patterns of SCC deficits as identified by scHIT (Figure 2), likely reflecting variable combination of peripheral and central involvement. In the first type, there are ipsilesional pan-SCC deficits, i.e., involving HC, AC, and PC, and contralesional SCC deficit involving HC, but not AC or PC. In the second, while there is variable bilateral HC involvement, vertical SCC is not affected. While both types are characterized by unilateral lesions causing bilateral HC impairment, which is probably explained by inhibitory floccular target neurons and interneurons between vestibular nuclei (7, 22), in the first type, ipsilesional vertical SCC is affected, suggestive of additional involvement of vestibular end organs/primary afferents/root entry zone, whereas in the second, lack of vertical SCC involvement suggests sparing of vestibular end organs and afferents. Horizontal catch-up saccade size is smaller when compared to acute peripheral vestibulopathy, despite similar HC impairment (7); the flocculus is possibly implicated since it modulates saccades (52) and an experimental lesion causes backward postsaccadic drift (53). CPA tumors expand progressively and cause compression of the brainstem and cerebellum, resulting in variable lesion of anatomical substrates that are similarly affected by AICA stroke.

### Gaucher Disease

Gaucher disease is a hereditary storage disorder due to glucocerebrosidase deficiency. Type 3 GD causes slowed saccades, more selective for horizontal than vertical (54), and vestibular impairments (8, 46). All SCC function is impaired, and due to saccade slowing, there is a paucity of catch-up saccades, most marked for HC. After a horizontal head impulse, the eyes may be “locked up” transiently until a resetting impulse in the opposite direction occurs. These findings may be accounted for by neuronal loss in the abducens and vestibular nuclei (55). If a patient with BVP does not consistently fixate on the center target, i.e., there would appear to be no catch-up saccade then this can potentially mimic GD. Repeated instruction to the patient and careful examination of saccade should minimize diagnostic confusion.

### Wernicke’s Encephalopathy

Acute Wernicke’s encephalopathy (WE), due to thiamine deficiency, may not present with the classic triad of encephalopathy, ataxia, and ophthalmoplegia (56). SCC deficit is characterized by selective, symmetrical HC impairment, with sparing of the vertical SCC, as demonstrated by both scHIT (47) and vHIT (10). Presumably, the medial vestibular nucleus, which receives afferents from HC, is particularly vulnerable to the effect of thia-mine deficiency (57). Prompt recognition and treatment not only restores SCC deficit (58) but also potentially prevent permanent neurological impairment. The major differential consideration of acute WE is AICA stroke presenting isolated bilateral HC deficit, and MRI is likely to provide additional diagnostic clarification.

### Cerebellar Degeneration

A characteristic abnormality of simultaneous cerebellar and vestibular involvement is the impaired visually enhanced vestibulo-ocular reflex (48), which may be present in certain types of cerebellar degeneration. This sign, along with gaze-evoked nystagmus, is of particular diagnostic importance as AC function sparing may not occur in cerebellar degeneration. In Friedreich’s ataxia (FA), SCC function is globally reduced (9), whereas in spinocerebellar ataxia type 6 (SCA6), SCC function may be increased or decreased depending on disease severity (49). The reason for this difference is not known, but is potentially related to anatomical substrate and pathogenesis: in FA, vestibular end organs and afferents are involved, whereas in SCA6, increased function is thought to be related to initial disinhibition of deep cerebellar nuclei due to cerebellar long term depression in suppressing Purkinje cell activity and decreased in function due to later neuronal loss in the flocculus. Cerebellar ataxia, neuropathy, and vestibular areflexia syndrome (CANVAS) is a sensory ganglionopathy (59), which causes bilateral HC and vertical SCC deficits (16). Both CANVAS and FA are associated with impaired visually enhanced vestibulo-ocular reflex and bilateral SCC deficits not sparing AC function.

## Conclusion

A number of central lesions can mimic BVP and poses diagnostic dilemma. Correct identification of central lesions is important, as both prognosis and treatment differ from BVP. In central lesions, specific patterns of SCC deficit may provide diagnostic clue and horizontal catch-up saccade size does not always correlate with SCC function. While scHIT remains the gold-standard for assessment of individual SCC function, the availability of vHIT has allowed clinicians to rapidly evaluate individual SCC function at point of care. In general, neurology practice vHIT availability is likely limited and clinical or bedside HIT remains a good screening test. Combined central and peripheral lesions are perhaps the most challenging to diagnose, and other eye movement abnormalities and MRI findings may be required to establish the diagnosis.

## Author Contributions

LC designed the study, analyzed eye movement data, and prepared and interpreted all the research data and figures. He was principally in charge of drafting, revising for intellectual content, and submitting the manuscript. GMH assisted in designing the study, interpreted research data figure, and in revising the manuscript for intellectual content.

## Conflict of Interest Statement

LC reports no disclosures. GH is an unpaid consultant to GN Otometrics, Taastrup, Denmark, but has received support from GN Otometrics for travel and attendance at conferences and workshops.
